# Supplementary material for: Hazardous Waste Management of Buffing Dust Collagen
Source: Materials (Basel). 2020 Mar 25;13(7):1498. doi: 10.3390/ma13071498 (PMC7177460; doi:10.3390/ma13071498)
Supplement: Supplementary file 1 [file materials-13-01498-s001.pdf]

materials

Supplementary Materials

Hazardous Waste Management of Buffing Dust Collagen

Miroslawa Prochon *, Anna Marzec and Oleksandra Dzeikala

Institute of Polymer and Dye Technology, Faculty of Chemistry, Lodz University of Technology, Stefanowskiego 12/16, 90-924 Lodz, Poland
* Correspondence: miroslawa.prochon@p.lodz.pl

Table S1. BDC Dust characteristic characteristics.

SAMPLES	Elemental Analysis Pract. (teor.) (%)			IR ν_{max} (cm^{-1})						Thermal Stability			
	Cr (Cr_2O_3)	N	S	ν(O–H)	ν(-CH_2-) (CH_3)	$\nu_{as}(COO^-)$	ν(C=O)	ν(-C-O)	ν(Cr-O)	T_5 (°C)	T_{50} (°C)	U_{150} (%)	P_{800} (%)
Dust-BDC	4.48	8.15	3.26	3461	2853	1410	1654	1345	528	80	310	7.8	12
-	Isoelectric point (IEP) (-)				Particle size (nm)		The size of the fraction with the largest numerical share (nm)				%- Number (%)		
Dust-BDC	5.9				295–664		469				31		

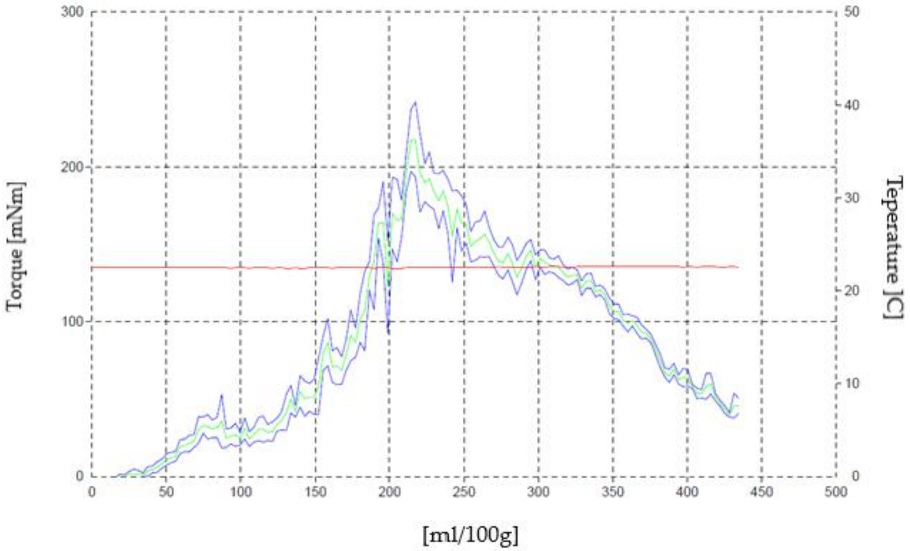

Figure S1. BDC Oil number (the sample weight was 4.3 g at 125 rpm and the dosing frequency was 4.0 mL/mL).
